# Neuro Emotional Technique as a Treatment for Separation Anxiety Related to Prenatal Stress: A Case Report

**DOI:** 10.7759/cureus.83558

**Published:** 2025-05-06

**Authors:** Peter Bablis, Ryan R Day, Sophia R Bablis, Henry Pollard

**Affiliations:** 1 Complementary Medicine, University Research Institute of Maternal and Child Health and Precision Medicine, Athens, GRC; 2 Complementary Medicine, Universal Health, Sydney, AUS; 3 Psychology, University of Technology Sydney, Sydney, AUS; 4 Research and Development, Universal Health, Sydney, AUS; 5 Physical Medicine and Rehabilitation, Faculty of Health Sciences, Durban University of Technology, Durban, ZAF

**Keywords:** childhood anxiety treatment, early life stress (els), epigenetic transmission, intergenerational trauma, mind-body intervention, neuro emotional technique (net), prenatal stress programming (pnsp), separation anxiety disorder, somatic memory, spence children’s anxiety scale (scas)

## Abstract

Prenatal maternal stress (PNMS) is increasingly recognised as a contributor to early-life emotional dysregulation through mechanisms such as prenatal stress programming (PNSP). Separation anxiety disorder (SAD) is the most common childhood anxiety condition, with limited treatment options for cases linked to prenatal factors. Neuro Emotional Technique (NET) is a mind-body intervention that targets unresolved emotional stress patterns through physiological and semantic integration. While used clinically in stress-related disorders, NET’s application in treating anxiety rooted in prenatal stress is underreported. An eight-year-old girl presented with severe separation anxiety, persistent nightmares, generalised anxiety, oppositional behaviour, and sleep difficulties. The onset of symptoms was hypothesised to be linked to PNMS. Previous interventions, including child psychology and natural remedies, were ineffective. The patient was treated with 11 NET sessions over six weeks. The Spence Children’s Anxiety Scale (SCAS) was utilised before and after treatment to assess symptom severity. NET treatment focused on identifying and integrating somatically stored emotional patterns, including in utero experiences. The “Somatic Imprint Model” was developed to conceptualise this process, amalgamating concepts we call somatic memory, stress imprinting, emotional conditioning, and trauma echoes. SCAS scores decreased by 25 points (child report) and 17 points (parent report). Improvements were noted in sleep, emotional regulation, independence, and confidence. The treatment was well tolerated with no adverse events. This case suggests that NET may offer therapeutic benefit in children with anxiety linked to prenatal stress, particularly when conventional approaches have been unsuccessful. While causality cannot be inferred from a single case, the magnitude of change observed warrants further investigation through controlled studies examining NET’s efficacy and mechanisms in early-life stress contexts.

## Introduction

Prenatal maternal stress (PNMS), broadly defined as psychological or physiological stress experienced during gestation, is highly prevalent, affecting 5.5-78% of pregnancies depending on definitions and measurement criteria [1,2]. PNMS has been associated with disruptions in the development of the fetal hypothalamic-pituitary-adrenal (HPA) axis [3,4], increasing vulnerability to a range of behavioural, emotional, and neuropsychiatric disorders across the lifespan through a mechanism referred to as prenatal stress programming (PNSP) [5,6].

The process of PNSP is thought to occur through stress-induced hormonal, immune, and epigenetic changes that influence fetal brain development [7]. Elevated maternal cortisol and inflammatory markers can cross the placental barrier, potentially altering neurodevelopmental pathways that govern stress reactivity and emotional regulation [8], predisposing to future psychopathology [9].

Among offspring exposed to PNMS, elevated anxiety symptoms are common [10-12]. Anxiety disorders often emerge early in life, with approximately half of all lifetime cases diagnosed before the age of eleven [13]. Separation anxiety disorder (SAD), characterised by persistent and developmentally inappropriate distress regarding separation from primary attachment figures, is the most frequently diagnosed childhood anxiety disorder [14]. While some degree of separation anxiety is typical in early development up to approximately three years of age [15], SAD reflects a pathological persistence of this response, interfering with social, academic, and family functioning [16,17].

Cognitive Behavioural Therapy (CBT) is widely regarded as the first-line treatment for SAD, demonstrating a responder rate of approximately 60% in clinical studies [18,19]. However, a substantial proportion of cases (approximately 40%) demonstrate incomplete or non-sustained recovery following conventional therapy, underscoring the need for adjunctive or alternative therapeutic approaches [19].

Emerging frameworks in clinical neuroscience suggest that emotional dysregulation linked to early life adversity, including prenatal exposures, may be more effectively addressed by interventions targeting physiological stress imprinting and mind-body integration [20]. These frameworks propose that unresolved emotional stress may be ‘stored’ in the nervous system through associative learning mechanisms, even when the original stressor occurred during pre-conscious developmental periods such as gestation [21].

Neuro Emotional Technique (NET) is a biopsychosocial intervention that integrates principles of cognitive neuroscience, traditional Chinese medicine, behavioural psychology, and psychophysiology into a structured 15-step clinical protocol [22,23]. NET posits that unresolved emotional experiences, whether conscious or non-conscious, can become associated with specific somatic, autonomic, and neurological patterns through conditioned learning and neuroplastic encoding. Using manual muscle testing as a real-time measure of physiological response to emotional stimuli, NET facilitates the identification of specific "neuro-emotional complexes" (NECs) and applies brief corrective interventions to promote reintegration of unresolved stress responses at a physiological level [22,23]. Unlike purely cognitive approaches, NET is theorised to target the somatic and autonomic imprint of unresolved stress patterns, offering a pathway to address trauma and dysregulation that may not be accessible through conscious recall or traditional talk therapy.

Clinical studies suggest that NET may be effective in reducing stress-related symptomatology in both adult and paediatric populations across a range of conditions, including anxiety, trauma-related symptoms, and behavioural dysregulation [23-32]. One case report has specifically documented NET's application in the management of childhood separation anxiety [33]; however, the technique's potential role in addressing anxiety symptoms linked to prenatal maternal stress exposures remains largely unexplored.

This case report presents a clinical example of NET applied to the treatment of separation anxiety in an eight-year-old female patient, with presenting symptoms suggestive of a stress imprint originating duringin utero development. It offers a novel perspective on how early life stress (ELS) may manifest behaviourally in childhood and how such patterns may be therapeutically accessed and resolved through an integrated mind-body approach.

## Case presentation

An eight-year-old female child presented with symptoms consistent with SAD, including intense fear of separation from her mother, difficulty sleeping alone, persistent nightmares, heightened generalised anxiety, emotional dysregulation, low self-esteem, and oppositional behaviour. She resisted bedtime, had difficulty falling asleep alone, and experienced recurring nightmares of being chased or consumed. She was often unable to remain at school for a full day due to distress about being apart from her mother. Her parents also expressed concerns regarding possible dyslexia and attention-deficit/hyperactivity disorder (ADHD).

The patient was born following a complicated delivery. Her mother experienced significant stress during pregnancy (rated 8/10 retrospectively on a Visual Analogue Scale (VAS) where a score of zero indicated no stress) that required prolonged postpartum bed rest. Formula feeding was introduced at two months of age due to poor milk supply, and mother-infant bonding was disrupted. Psychosocially, she experienced early sibling rivalry and parental conflict. Her past medical history included recurrent ear infections and a tonsillectomy at age five.

Prior interventions included medical counselling and paediatric CBT, as well as trialling off-the-shelf multivitamin supplements for children. These interventions were reportedly ineffective in alleviating the child’s anxiety-related symptoms. The patient's general practitioner provided documentation listing learning difficulties, behavioural concerns, and generalised anxiety. No medication had been prescribed at the time of NET referral, nor were any medications used during the NET treatment period.

Clinical findings

Presenting Symptoms and Behaviours

At the initial consultation, the patient presented with high levels of generalised and separation anxiety, as reported by her parents and by clinical observation. She exhibited intense distress at the prospect of separation from her mother, including clinginess, tearfulness, and somatic complaints (e.g., stomach aches) when preparing for school or bedtime. Her sleep was severely disrupted by frequent nightmares and resistance to falling asleep alone, which she often delayed through avoidant behaviours, such as stalling tactics, becoming clingy or panicked, and intentionally trying to stay awake.

Behaviourally, she demonstrated oppositional tendencies, difficulty following instructions, emotional outbursts, and frequent frustration in response to minor environmental changes. Her affect was often flat or anxious, with a low tolerance for uncertainty or transition.

These findings were corroborated by parent reports and directly observed during treatment sessions. While parent reports are subject to potential bias, the consistency between parent observations, patient self-report, and practitioner observations strengthens the validity of the clinical impressions. The patient also displayed signs of hypervigilance and over-reactivity, suggestive of heightened stress sensitivity, which became central to the formulation of treatment targets during NET sessions. Physical examination revealed no neurological or structural abnormalities.

Diagnostic Tool and Testing

The Spence Children’s Anxiety Scale (SCAS) is a validated tool for assessing anxiety symptoms in children and adolescents [34-36], with child self-report and parent-report formats available for different age groups [37,38]. The SCAS is considered psychometrically robust, demonstrating strong internal consistency [39], test-retest reliability [38], and discriminant validity for anxiety disorders [40,41]. Normative data used in this report were drawn from an Australian sample of 2,000 school-aged children [38].

The SCAS includes six subscales: Panic Attack and Agoraphobia (PAA), Separation Anxiety (SA), Physical Injury Fears (PIF), Social Phobia (SP), Obsessive-Compulsive Behaviours (OC), and Generalised Anxiety Disorder (GAD) [42,43]. Higher scores indicate greater symptom severity, with a total score range of 0-114 [44,45]. A change of 14 points or more in total score is considered clinically significant [46]. Raw scores are converted into t-scores based on age and gender, allowing standardised interpretation. A t-score of 50 represents the population mean; scores ≥60 are considered elevated, and scores ≥70 reflect the top 2% of severity [47]. SCAS questionnaires were completed at baseline and after six weeks of NET treatment by both the child and her mother.

We used both t-scores and percentiles to contextualise changes in symptom severity. The results from all four assessments (two child reports, two parent reports) were tabulated and analysed using published scoring protocols [48].

A timeline of events outlining major developmental, medical, psychosocial, and therapeutic events relevant to the patient’s presentation of separation anxiety, beginning from birth through to post-treatment outcomes, is provided in Table 1.

**Table 1:** Timeline of key symptoms and events Key milestones include early life stress exposures, initial symptom emergence, previous interventions, formal diagnoses, commencement of Neuro Emotional Technique (NET) treatment, and subsequent clinical improvements tracked over six weeks. SCAS: Spence Children's Anxiety Scale

Time Point	Event	Remarks
Birth–Infancy	Difficult birth, formula feeding	Maternal complications disrupted bonding and contributed to early stress
Early Childhood	Family stress, sibling rivalry	Contributed to emotional insecurity and sensitivity to change
Age 5 years	Tonsillectomy	Medical history, recurrent ear infections
Age 6–7 years	Failed interventions	Brief child psychology and supplements—no improvement
Age 8 years (Baseline)	NET intake, SCAS assessment	Severe separation anxiety, nightmares, school refusal, inability to adapt to change
Week 1	NET treatment initiated	Early improvements (e.g., willingness to sleep alone and a reduction in her fear of the dark)
Week 4	Parent follow-up	Reported 50% symptom improvement
Week 6	SCAS post-test + school feedback	SCAS scores dropped ≥14 points; improved sleep, confidence, and behaviour

Therapeutic intervention

NET is a mind-body approach used by certified practitioners that combines elements of traditional Chinese medicine, semantic inquiry, and applied neuroscience to address unresolved emotional stress. NET uses muscle testing as a biofeedback mechanism to identify emotionally-charged memories or patterns that are stored somatically and influence present behaviour. Once identified, specific interventions are applied to integrate the emotional pattern and reduce the associated physiological stress response [22].

The Somatic Imprint Model (SIM)

**Figure 1:**
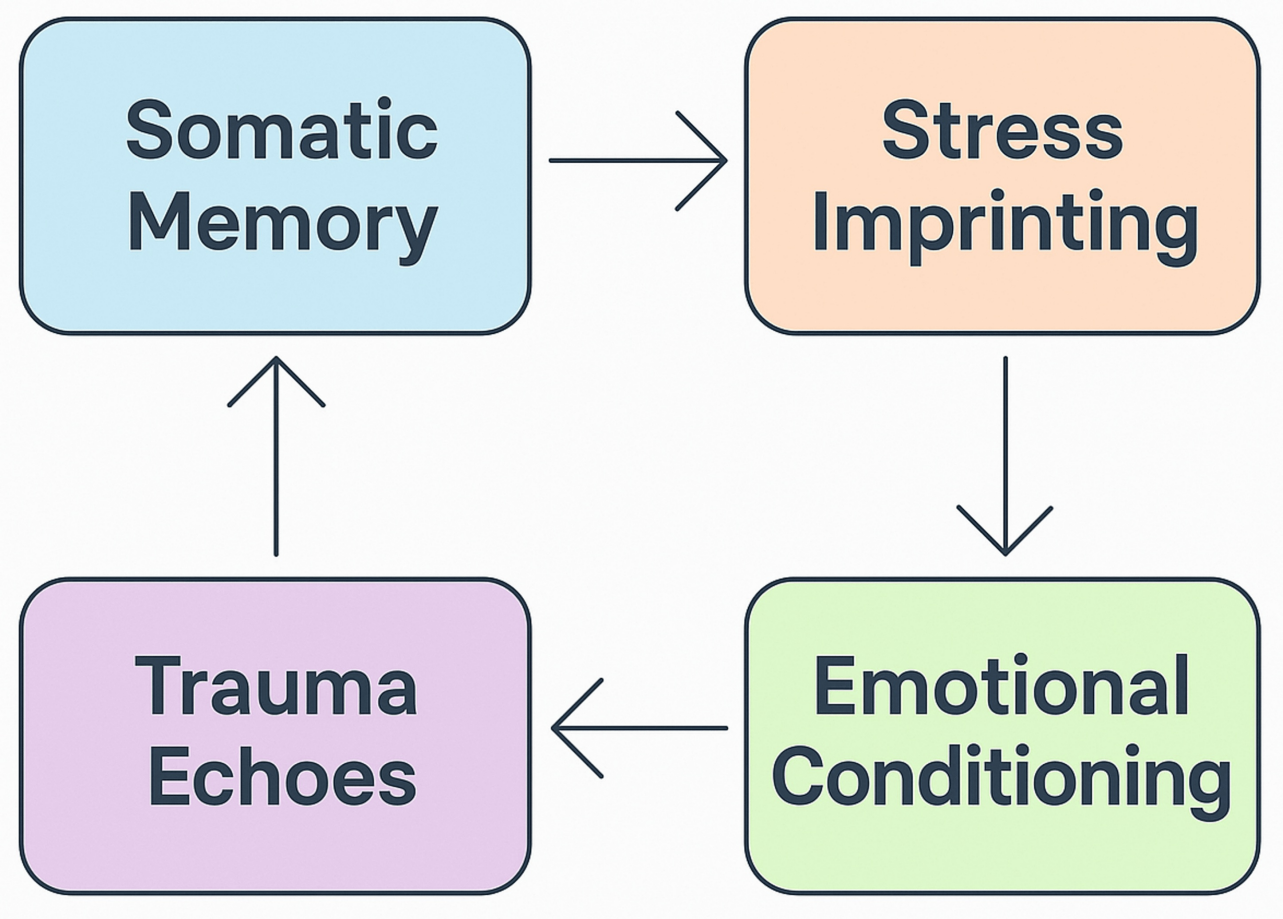
The Somatic Imprint Model: cyclical dynamics of somatic memory, stress imprinting, emotional conditioning, and trauma echoes

To provide a theoretical framework for understanding NET’s impact in cases of prenatal stress, we developed the SIM (Figure 1), which illustrates how early stressful experiences such as PNMS can initiate somatic memory and contribute to stress imprinting at the physiological and emotional levels. Through associative learning and neuroplastic encoding, environmental cues become conditioned triggers (emotional conditioning), leading to recurring emotional and behavioural responses termed trauma echoes. These echoes reinforce somatic memory, creating a cyclical pattern that can perpetuate dysregulation, unless therapeutically addressed.

The SIM integrates the four core mechanisms based on recognised concepts found in the scientific literature:

Somatic memory: Emotional and physiological experiences, especially those encoded prenatally, can be stored non-consciously through limbic system pathways due to the immaturity of the prefrontal cortex [6,21].

Stress imprinting: Maternal trauma and/or significant stress during pregnancy can alter fetal development [49,50] via epigenetic regulation and HPA axis programming [51,52], with evidence of intergenerational transmission of stress-related markers [53,54].

Emotional conditioning: Through contextual binding, associative learning, and Pavlovian conditioning, environmental stimuli paired with maternal stress can become conditioned within neural circuits through experience-dependent neuroplasticity, establishing non-conscious triggers of fear [55,56] and dysregulation later in life [57,58].

Trauma echoes: Drawing from psychoanalytical frameworks such as repetition compulsion and unresolved emotional schemas [59], preverbal trauma may be re-enacted through repetitive behavioural, emotional, or somatic patterns [60,61], a mechanism commonly observed in intergenerational trauma transmission [62].

This model facilitated the interpretation of NET treatment outcomes in the present case.

NET Application in this Case

NET was applied in 11 sessions of approximately 15 minutes each, over a six-week period. Each session involved identifying stress patterns using muscle testing and verbal emotional cues, with physiological integration techniques to resolve dormant imprints. Below are two illustrative examples:

Example 1. Nighttime fear and separation anxiety: The patient presented with intense separation anxiety, refusal to sleep alone, fear of the dark, and recurring nightmares involving pursuit and entrapment. NET inquiry revealed an underlying emotional theme of “impending doom,” traced to a maternal trauma during pregnancy, specifically, an emergency hospital admission where the mother feared for her baby’s survival.

This experience likely encoded a somatic memory of existential threat during fetal development. The accompanying surge in maternal glucocorticoids may have contributed to stress imprinting via HPA axis programming [7,8]. Over time, the child’s fear of the dark and aversion to sleeping alone appeared linked to emotional conditioning, whereby the sensory cue of darkness acted as a contextual trigger that unconsciously activated/intensified a neurophysiological fear response, consistent with her being physically in the darkness of the womb during her mother’s traumatic experiences. Furthermore, her separation anxiety appeared to be contextually bound negatively to the traumatic separation from her mother during her life-threatening birth process. In turn, these contextual triggers activated the body's innate survival-oriented stress system [63], mediated in part by the amygdala, a key structure in the limbic system responsible for detecting threat and initiating reflexive protective autonomic and behavioural reactions [64-66].

These reactions persisted as trauma echoes, surfacing through maladaptive behaviours such as avoidance, fear of the dark, and separation anxiety. Within days of NET treatment, she began sleeping alone consistently, and her nightmares and fear of the dark significantly diminished, suggesting resolution of the underlying somatic pattern.

Example 2. Reactivity to unpredictable events: The child exhibited heightened emotional reactivity to unplanned events, often escalating into aggressive or fearful outbursts. Using NET, the practitioner traced this reactivity to a somatic memory formed in utero-specifically, fetal exposure to the emotional distress associated with an unplanned conception. At the time, the parents reported experiencing significant psychosocial stress and emotional upheaval, which led to moments of uncertainty about whether to proceed with the pregnancy. While the decision was ultimately made to continue the pregnancy, the absence of joy and the presence of maternal anxiety appeared to imprint as a pervasive sense of existential threat for the developing fetus.

This emotional environment may have established a stress imprint in the fetal nervous system, contributing to the child's elevated baseline arousal and increased vigilance. In particular, her later need for predictability and control may have stemmed from this early programming. It is hypothesised that the fetus associated the mother's stress response with the discovery of her pregnancy, a core unpredictable event, and, via contextual binding, began to subconsciously pair future unpredictability with threat.

During the current period in the child's life, benign stimuli such as last-minute schedule changes triggered physiological responses disproportionate to the situation. This was understood as emotional conditioning, where unpredictable events had become contextually linked to an unresolved imprint of prenatal existential distress. The repetitive behavioural responses observed, rigid control, defiance, and meltdowns, were reflective of trauma echoes, suggesting the child was non-consciously reenacting persistent pre-verbal stress/threat responses to perceived environmental threats.

Following targeted NET sessions addressing these themes, the child demonstrated markedly reduced sensitivity to change, improved emotional regulation, and increased flexibility in daily routines, along with a growing internal sense that change could be safe and manageable.

Follow-up and outcomes

Response to Treatment

Over a six-week treatment period, the patient received 11 NET sessions, similar to those described in the examples above. The treatment was well tolerated, with no signs of distress or discomfort during sessions. No adverse events or negative reactions were reported.

After the first treatment (illustrated in Example 1 above), the child was independently initiating sleep without distress and no longer required co-sleeping. Her mother reported other observable improvements within the first two weeks, including reduced nighttime comfort eating, less resistance at bedtime, and improved mood. Within four weeks, emotional regulation, confidence, and attention span had noticeably improved according to both parents and her schoolteacher, who was not informed of the intervention.

At the six-week mark, the SCAS was re-administered to both the patient and her mother. The child’s total SCAS score [47, 67] decreased by 25 points (from the 82nd to 29th percentile), and the parent-reported score [68] decreased by 17 points (from the 67th to 29th percentile). Both exceeded the 14-point threshold for clinically significant change [46], reflecting a shift from clinically elevated symptoms to a score within the average range for age and gender-matched peers, and indicating a meaningful reduction in symptom severity.

**Figure 2:**
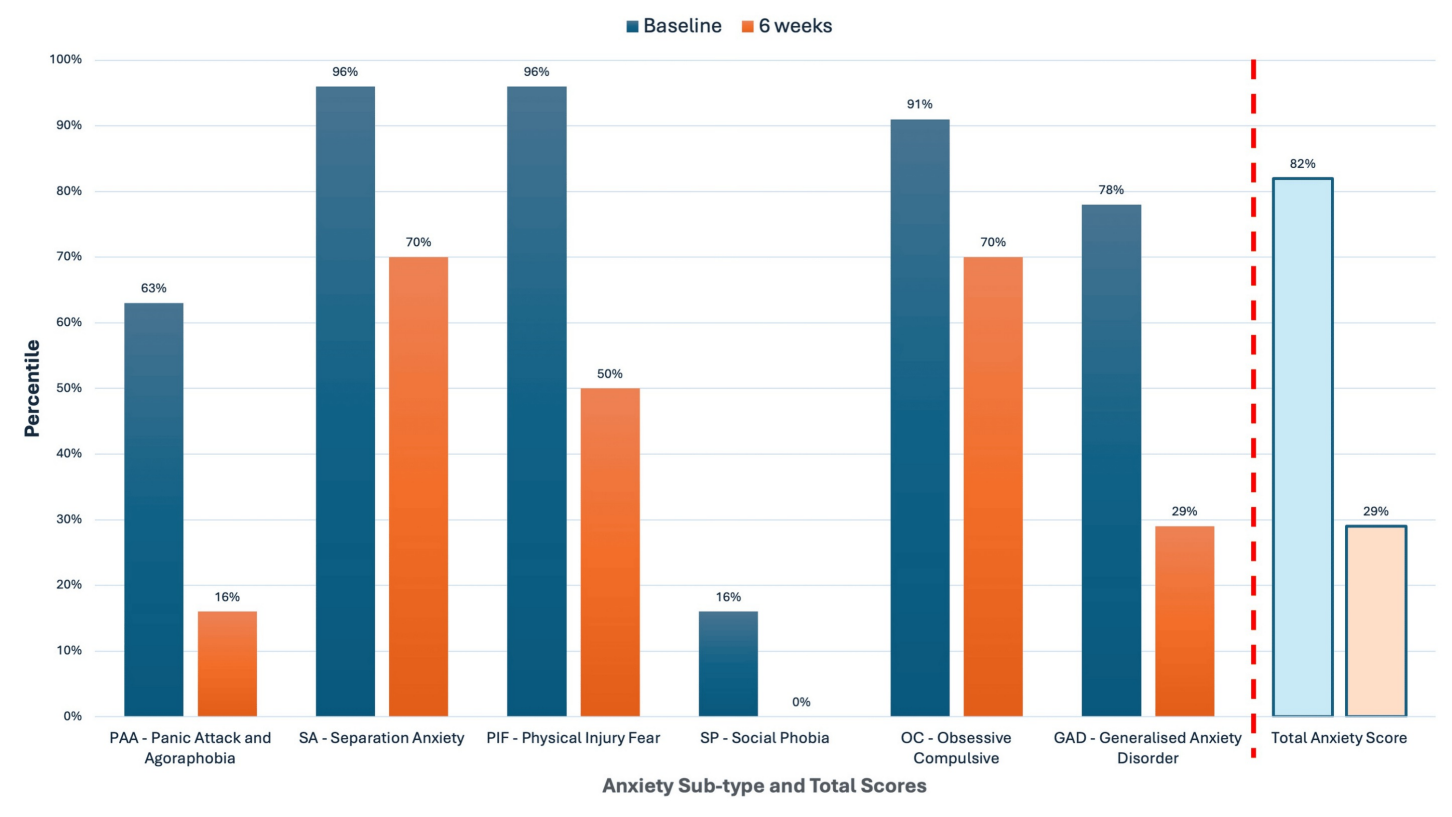
Child Self-Report (SCAS-C) results before and after NET treatment Percentile scores are shown at baseline (dark blue) and after six weeks of NET intervention (orange). The six anxiety sub-types assessed are Panic Attack and Agoraphobia (PAA), Separation Anxiety (SA), Physical Injury Fear (PIF), Social Phobia (SP), Obsessive-Compulsive (OC), and Generalised Anxiety Disorder (GAD). The Total Anxiety Score is displayed separately to the right of the red dotted line, which visually separates the anxiety sub-types from the total score. A substantial reduction across all subtypes and the total anxiety score was observed following treatment. Percentile scores are based on normative data for age- and gender-matched Australian children [38]. A decrease in percentile rank indicates a relative reduction in symptom severity compared to the population average. NET: Neuro Emotional Technique; SCAS-C: Spence Children’s Anxiety Scale child's report

**Figure 3:**
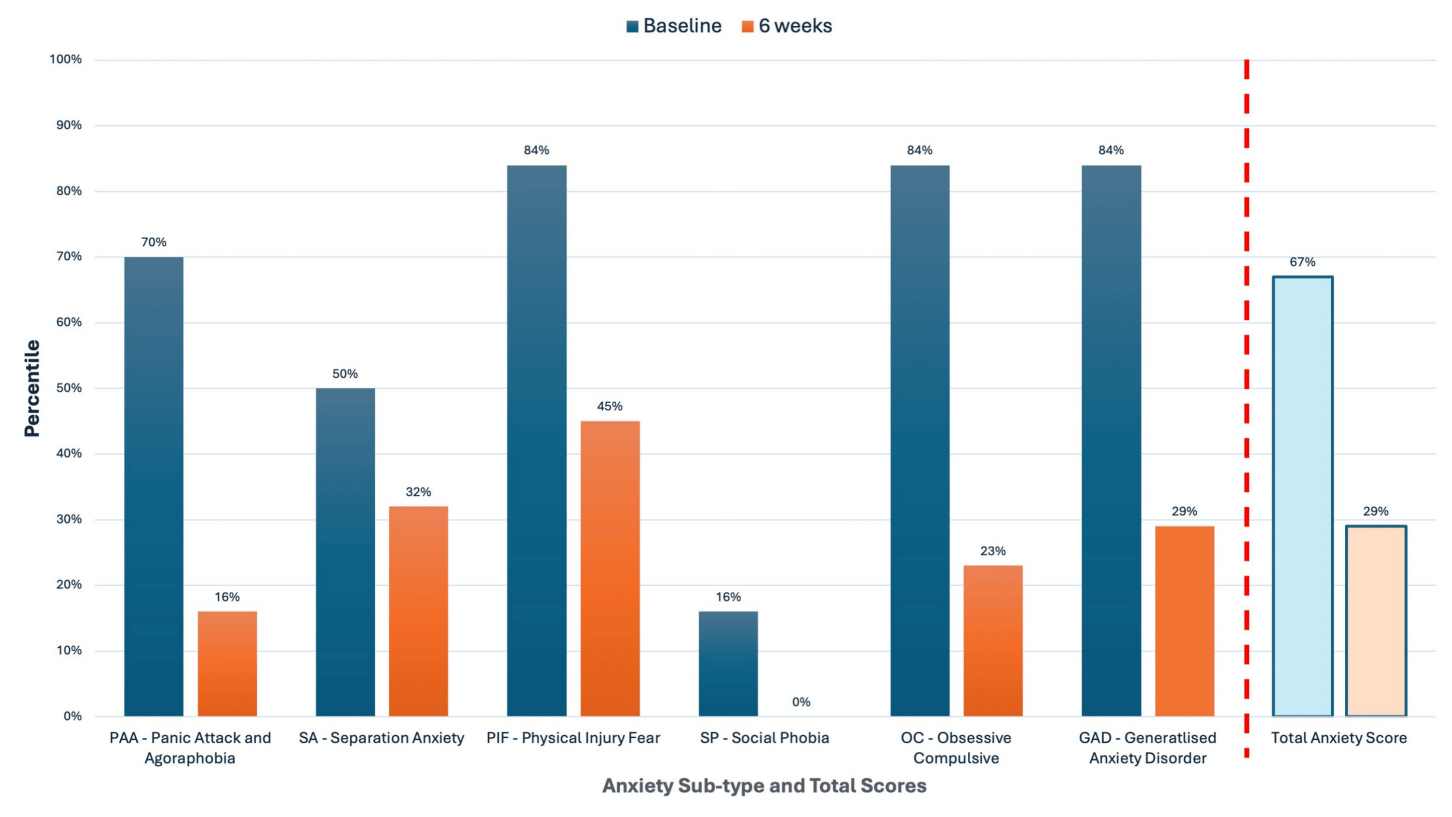
Parent Report (SCAS-P) results before and after NET treatment Percentile scores based on the parent's observations are shown at baseline (dark blue) and after six weeks of NET treatment (orange). Anxiety sub-types include Panic Attack and Agoraphobia (PAA), Separation Anxiety (SA), Physical Injury Fear (PIF), Social Phobia (SP), Obsessive-Compulsive (OC), and Generalised Anxiety Disorder (GAD). The Total Anxiety Score is displayed separately to the right of the red dotted line, which serves as a visual divider between the sub-type scores and the overall anxiety score. Consistent improvements were reported across all domains after treatment. Percentile scores are based on normative data for age- and gender-matched Australian children. A decrease in percentile rank indicates a relative reduction in symptom severity compared to the population average. NET: Neuro Emotional Technique; SCAS-P: Spence Children’s Anxiety Scale parent report

Improvements were evident across all six anxiety subscales. For example, the SCAS separation anxiety subscale score decreased from the 96th to the 70th percentile in the SCAS child's report (SCAS-C) and from the 50th to the 32nd percentile in the parent's report (SCAS-P). Similar percentile reductions were observed in generalised anxiety, panic attacks, and physical injury fears, suggesting broad-based improvements in internalising symptoms. The quantitative changes in anxiety sub-types and total scores, as assessed by both child and parent reports, are illustrated in Figure 2 and Figure 3.

The reduction in separation anxiety symptoms, as reflected on the SCAS-C, may correlate with improved functional outcomes that were observed, such as increased independence at bedtime and reduced anticipatory distress when separating from caregivers. These improvements align with parental reports of more settled sleep initiation and greater emotional flexibility in previously triggering situations.

## Discussion

This case report presents a rare example of clinically significant anxiety symptoms in a child whose presentation appears to be more strongly linked to PNMS than to her own lived experiences. The intervention, NET, was associated with improvements across behavioural, emotional, and psychometric domains, including a 25-point reduction in the child’s SCAS total score.

PNMS refers to stressors experienced during pregnancy, including trauma, hardship, and emotional distress, and has been associated with long-term impacts on behaviour, cognition, and emotional regulation in children [69,70]. These effects are often understood through the lens of PNSP, a form of ELS [71,72], which derives from the Developmental Origins of Disease framework [73]. PNSP suggests that fetal adaptations to maternal distress, such as heightened glucocorticoid exposure, can alter developmental trajectories, particularly via the HPA axis [7,8]. If not integrated or extinguished, these adaptations can persist postnatally, shaping physiology, emotional regulation, and health trajectories across one’s lifespan [74-76].

Excess glucocorticoids during sensitive periods of fetal development may disrupt fetal brain maturation and stress regulation, via dysregulation of the Psycho-Immune-Neuro-Endocrine (PINE) network and energy systems [77,78], with potential downstream consequences for anxiety and mood disorders [3,79]. These processes are central to the SIM, which proposes that emotionally salient experiences, even in the absence of conscious memory, may be encoded somatically/physiologically and later reactivated by environmental or emotional cues.

The timing of maternal stress appears to be a key variable, with certain windows of gestation carrying heightened vulnerability [80,81]. For instance, stress exposure later in pregnancy has been associated with increased cortisol reactivity and illness risk in children [4,82]. While not all children exposed to PNMS develop anxiety, mounting evidence suggests that such exposures increase susceptibility to emotional dysregulation, particularly when early nurturing or postnatal support is insufficient [83,84].

Another plausible mechanism of transmission is epigenetic programming, whereby maternal distress alters fetal gene expression via mechanisms like DNA methylation and histone modification [85,86]. These changes can shape stress reactivity, emotional regulation, and neurodevelopmental sensitivity [87,88]. Intergenerational studies of trauma survivors and their offspring have reported changes in biological aging and increased post-traumatic stress disorder (PTSD) susceptibility, even in the absence of direct trauma [89].

In this case, NET was used to target emotional responses hypothesised to stem from unresolved imprints of prenatal stress. The treatment protocol aligns with emerging views that emotional and physiological patterns may be encoded somatically and influence behaviour long after the original event. Using the SIM, which integrates somatic memory, stress imprinting, emotional conditioning, and trauma echoes, NET may offer a way to identify and resolve these patterns without requiring conscious memory or verbal recall.

Comparison with other therapies

CBT remains the first-line treatment for childhood anxiety, including SAD, with response rates of approximately 60% across multiple studies [18]. Pharmacotherapy, particularly selective serotonin reuptake inhibitors (SSRIs), can also be effective but may produce short-term adverse effects, including gastrointestinal symptoms and sleep disturbance, particularly in younger children [90,91].

Alternative or adjunctive therapies have demonstrated promise. Parent-Child Interaction Therapy has shown efficacy in treating SAD in younger children [92,93], and Child-Centred Group Play Therapy has been effective in reducing separation-related distress in preschoolers [94]. More intensive or trauma-focused approaches are sometimes recommended for complex or treatment-resistant cases [95].

In contrast, NET represents a novel, non-pharmacological intervention that targets subconscious, somatically encoded emotional responses, especially those linked to prenatal or preverbal experiences. While the evidence base for NET in paediatric anxiety is limited, this case suggests it may offer benefit where conventional approaches are ineffective or poorly tolerated, warranting further research.

In this case, the observed generalised anxiety reductions suggest that the intervention may have had a broader regulatory effect beyond the primary presenting symptom. This raises the possibility that NET, by resolving underlying stress imprints, may simultaneously alleviate comorbid anxiety domains. The multidimensional changes also suggest that NET may support both behavioural and physiological regulation in cases where anxiety is linked to prenatal or preverbal emotional stress exposures.

While the single-case design limits generalisability, the magnitude of improvement, consistency across multiple domains, and use of validated psychometric measures collectively support the need for further investigation of NET’s potential applications in prenatal stress programming and intergenerational trauma contexts.

Strengths and limitations

This case report contributes to a relatively unexplored clinical domain, childhood SAD potentially rooted in PNMS, by documenting the use of NET as a therapeutic intervention. The SIM, developed by the authors to assist with the interpretation of this case, alongside integration of evidence on intergenerational trauma and PNSP, provides a meaningful theoretical framework for further research. The use of a validated outcome measure (SCAS), supported by parent report and school observations, strengthens the internal validity of the findings.

However, the single-case design limits generalisability and precludes causal inference. The absence of randomisation and long-term follow-up, as well as the potential influence of placebo effects or natural symptom remission, further constrain the conclusions that can be drawn. Reliance on subjective observations, while informative in a clinical setting, introduces potential bias.

This report does not imply that all children exposed to PNMS will develop anxiety, nor does it suggest that NET is universally effective. Resilience factors such as secure attachment, postnatal nurturing, and environmental support may mediate outcomes and should be considered in future studies.

To evaluate NET’s efficacy and mechanism of action more robustly, future research should include larger observational studies, randomised controlled trials, and investigations into neurobiological and epigenetic outcomes. Comparisons with established therapies may lead to multimodal treatment approaches for paediatric anxiety linked to prenatal stress.

Patient perspective

The child’s mother described the treatment as transformative, stating: “For the first time, my daughter could sleep alone, go to school without tears, and enjoy her day. It changed everything for our family.” She reported noticeable improvements in her daughter’s confidence, emotional balance, and ability to cope with everyday challenges.

## Conclusions

This case provides preliminary observations highlighting the potential of NET as a novel adjunctive intervention for childhood separation anxiety, particularly when symptoms may be linked to PNMS. The child demonstrated rapid, multidimensional improvement following targeted NET treatment, suggesting a possible role for interventions that address the emotional imprints of in utero stress in selected cases, especially when conventional approaches fail. These findings offer preliminary, anecdotal support for the hypothesis that unresolved prenatal emotional experiences can contribute to anxiety via various stress programming mechanisms. By identifying and integrating these imprints, NET may provide a complementary approach to existing therapies, targeting preverbal stress patterns that are not easily accessed through cognitive or behavioural interventions. However, the single-case design limits generalisability, and caution must be exercised in interpreting the findings. Further research is essential to assess reproducibility and refine clinical applications. While the improvement observed in this case is notable, it is also possible that non-specific therapeutic effects, such as increased parental attention or expectancy, contributed to the outcome. Developmental variability and spontaneous remission cannot likewise be ruled out as contributing factors. Controlled trials, larger cohorts, and objective biological markers are recommended to evaluate NET’s potential role in mitigating the long-term effects of prenatal stress and enhancing outcomes in paediatric mental health care.
